# Immune Thrombocytopenic Purpura and Gastritis by H. pylori Associated With Type 1 Diabetes Mellitus

**DOI:** 10.7759/cureus.512

**Published:** 2016-02-24

**Authors:** Carlos Culquichicón-Sánchez, Ricardo Correa, Igor Flores-Guevara, Frank Espinoza Morales, Christian R Mejia

**Affiliations:** 1 Facultad de Ciencias de la Salud, Universidad Nacional de Piura, Piura, Perú; 2 NICHD, National Institute of Health; 3 Servicio de Diabetes, Instituto Cardiovascular Lezica; 4 Escuela de Medicina Humana, Universidad Continental, Huancayo, Peru

**Keywords:** type 1 diabetes mellitus, inmune thrombocytopenic purpura, idiophatic thrombocytopenic purpura, latin america, peru

## Abstract

We present the 15th case reported worldwide and 3rd case reported in Latin America of immune thrombocytopenic purpura associated with Type 1 diabetes mellitus in Scopus, MEDLINE, and SciELO. An 11-year-old male patient of mixed ethnicity with immune thrombocytopenic purpura, Type 1 diabetes mellitus, and gastritis due to *H.* *pylori* presented to the emergency room with petechiae, ecchymosis, and gingival and conjunctival bleeding that had been worsening for the past three months. The patient had a body mass index of 18.85 kg/m^2^ (P75). A biochemical analysis showed 1×10^9^ platelets/L, increased prothrombin time, increased partial thromboplastin time, and an HbA1C of 7.84% on admission. He was prescribed a single dose of intravenous methylprednisolone 750 mg in 100 mL of NaCl and daily oral 50 mg prednisolone, with intravenous 250 mg tranexamic acid every eight hours. The patient’s glycemic control was continued with the administration of insulin glargine (30 units every 24 hours) and prandial insulin glulisine (five to eight units per meal). Before admission, the patient was on a prescribed treatment of sitagliptin 50 mg and metformin 850 mg, but this was suspended in the emergency room. For the eradication of *H.* *pylori *he was prescribed amoxicillin 500 mg every eight hours, oral clarithromycin 335 mg every 12 hours, and IV omeprazole 40 mg. After 15 days, he showed disease resolution and he was discharged to his home with orders to follow-up with pediatrics, hematology, and endocrinology services. The first-line treatment for immune thrombocytopenic purpura patients with active bleeding and a platelet count < 30,000 platelets/μl is the administration of corticosteroids and inmunoglobulin.

## Introduction

Immune thrombocytopenic purpura (ITP), formerly known as idiopathic thrombocytopenic purpura, is an autoimmune bleeding disorder in which autoantibodies are formed against platelets, which are then destroyed by large-scale phagocytosis in the spleen and, to a lesser extent, in the liver. ITP is characterized by cutaneous, mucosal bleeding with variable manifestations among children and adults. The presentation is usually acute, secondary to a viral infection. It is a mostly self-limiting disease with an approximate six-month duration [1]. Type 1 diabetes mellitus (T1DM) is often associated with other autoimmune diseases such as thyroiditis, celiac disease, and vitiligo in a complex syndrome called autoimmune polyglandular syndrome (APS). The association with ITP is very rare [2]. Controlling *H.* *pylori,* a cosmopolitan pathogen, may offer an advantage in long-term glycemic control [3]. We present the 3rd Latin American and 15^th^ worldwide case of ITP associated with T1DM reported in Scopus, MEDLINE, and SciELO. Informed patient consent was obtained for this study.

## Case presentation

A thin 11-year-old male of mixed ethnicity with T1DM presented to the emergency room (ER) with a progressive history of petechiae and ecchymosis mainly in the lower extremities. The patient had been diagnosed with T1DM three months before admission to the ER and was on a regular treatment of a low calorie diet, once-daily glargine (30 units), and prandial insulin glulisine (five units after breakfast and dinner and eight units after lunch). The patient has a family history of dyslipidemia and obesity but no family history of autoimmune disease. 

The patient’s mother reported that his skin lesions were progressively appearing from January 2015, and were associated with polyuria, polydipsia, and “sweet” urine. The pediatrician performed a finger stick analysis that revealed a very elevated blood glucose level (500 mg/dL) and the patient was referred to an endocrinologist. The endocrinologist made the diagnosis of T1DM and started the patient on an insulin regimen as described above. The purpuric syndrome was associated with pallor, fatigue, and a general deterioration that intensified in April 2015, shortly before admission.

A month before the admission, the petechiae and ecchymosis were accentuated on the patient’s lower limbs in the absence of a traumatic antecedent. Fifteen days after, the lesions extended to the rest of the body and the patient started presenting dark brown stools with abnormal odor. One week before admission, the patient experienced a minor injury in the back of his right thigh, after which he started complaining of increased volume in the affected area along with a moderate intensity headache (described as a 6/10 on a numeric pain scale). The day of admission, he had ecchymosis on his lower lip after mild trauma, spontaneous bleeding of the gums, and bilateral conjunctival hemorrhage.

On April 14, 2015, the patient had a complete blood count (CBC) that showed pancytopenia that promptly resulted in hospitalization for further evaluation. The patient’s results from his physical examination are as follows: weight was 43 kg, height was 151 cm (both height and weight are between P75-P90), with a body mass index of 18.85 kg/m^2^ (P75). A mild skin pallor was noted along with multiple ecchymoses (2–5 cm diameter) on the upper limbs. Petechiae (< 2 mm diameter) were distributed symmetrically throughout the body but were predominantly located on the lower limbs. Non-pitting edema was noted on the back of the right thigh with mild pain on palpation and a hepatic height of 9 cm. No splenomegaly was found. The patient was given a single dose of intravenous (IV) methylprednisolone 750 mg in 100 mL of NaCl and IV tranexamic acid 250 mg every eight hours. Oral prednisolone 50 mg/day was continued. Two days after admission, the patient had a fever of 39°C accompanied by holocraneal headache of moderate intensity, which were treated with antipyretic agents and pain medications.

During his hospitalization, the patient was diagnosed with *Helicobacter pylori *gastritis via immunochromatography. The *H. pylori* gastritis was treated with oral amoxicillin 500 mg every eight hours, oral clarithromycin 335 mg every 12 hours, and IV omeprazole 40 mg. Given the patient’s T1DM, his glycemia was monitored before meals.

On admission, the patient’s platelets were 1x10^9^/L. At discharge, his platelets were 25x10^9^/L, and his prothrombin time and partial thromboplastin time were increased. A CBC reported moderate anemia, and liver function test results were normal. Immunological tests for dengue were negative. A total remission of purpura was evident, and the patient’s glycemic control was suitable. He was sent home with a prednisone taper regimen, an insulin and diet regimen, and orders to follow up with pediatrics, hematology, and endocrinology services.

## Discussion

There are few cases in the literature regarding the association of ITP with T1DM. ITP is more frequently found secondary to human immunodeficiency virus or viral hepatitis [4]. The current literature indicates an association of ITP and T1DM with sarcoidosis, pregnancy, varicella [5], polyglandular syndrome type III, megasigmoides, pernicious anemia [6], or Graves' disease [7] with a predominance in European and Asian populations. To our knowledge, this is the 3rd case in Latin America and the 15^th^ worldwide that has been reported in Scopus, MEDLINE, and SciELO.

An *H. pylori* infection exacerbates the difficulties of glycemic control for patients with T1DM [3], therefore, the treatment of the *H. pylori* infection becomes crucial for these patients. 

Recent research in the treatment of ITP established a close relationship with T1DM by the CD20 receptor expressed on the cell membrane of B-lymphocytes. Although T1DM is considered an autoimmune disease mediated by T-lymphocytes, B-lymphocytes also play an important role in the development of the disease by secreting specific autoantibodies against islet cells (used as disease markers), and behaving like cells’ antigen-presenting subpopulations of CD4 plus T-cells [8-10].

In fact, a report recently published demonstrated the efficacy of rituximab (a specific monoclonal antibody against the CD20 receptor) administered to a patient with a recent onset of T1DM suffering from ITP refractory to immunoglobulin, with high doses of steroids and cyclosporine. This regimen not only achieved ITP resolution but also allowed the patient to achieve glycemic control for 28 months without insulin therapy [11]. The findings of this isolated case support other studies [12] that have found rituximab use in newly diagnosed T1DM retards the deterioration of the beta cells, expressed as an increase in C-peptide levels and reduces the daily insulin dose required. This finding should encourage studies of greater magnitude to elucidate new therapeutic lines in this and other susceptible populations.

## Conclusions

In summary, the pathophysiology of the association between ITP and T1DM is unclear. However, we currently know beta cells play a role by secreting autoantibodies specific for islet cell proteins and acting as antigen-presenting cell populations for CD4 plus T-cells, thus the apparent efficacy of rituximab. We emphasize the importance of early erradication of *H. pylori* to allow proper long-term glycemic control. The first-line treatment for ITP patients with active bleeding and a platelet count < 30,000 platelets/μl is the administration of corticosteroids and immunoglobulin. Therefore, further investigation is needed to understand the etiology, clinical manifestation, complications, and risks groups in order to properly resolve a possible syndrome. 

## References

[1] Verdugo LP, Kabalan BP, Silva CR (2011). Clinical guidelines for the management of pediatric patients with primary immune thrombocytopenia (ITP) (Article in Spanish). Rev Chil Pediatr.

[2] Escobar-Jiménez F (2009). Clinical guidelines for diabetes mellitus type 1 (Article in Spanish). Endocrinol Nutr.

[3] Dai YN, Yu WL, Zhu HT (2015). Is Helicobacter pylori infection associated with glycemic control in diabetics?. World J Gastroenterol.

[4] Monteagudo E, Fernandez-Delgado R, Sastre A (2011). Protocol for the study and treatment of immune thrombocytopenic purpura (ITP). ITP-2010 (Article in Spanish). An Pediatr (Barc).

[5] Khadwal A, Deepthi N (2008). Severe thrombocytopenia in a child with type 1 diabetes. Pediatr Endocrinol Diabetes Metab.

[6] Kondo H, Imamura T (1999). Pernicious anemia (PA) subsequent to insulin-dependent diabetes mellitus and idiopathic thrombocytopenic purpura, and effects of oral cobalamin on PA [1]. Am J Hematol.

[7] Uptodate Database (2015). Uptodate database. Online.

[8] Gupta S (2012). Immunotherapies in diabetes mellitus type 1. Med Clin North Am.

[9] Silverman GJ, Weisman S (2003). Rituximab therapy and autoimmune disorders: prospects for anti-B cell therapy. Arthritis Rheumatol.

[10] Pescovitz MD, Greenbaum CJ, Krause-Steinrauf H (2009). Rituximab, B-lymphocyte depletion and preservation of beta-cell function. N Engl J Med.

[11] Von Laer Tschudin L, Schwitzgebel VM, von Scheven-Gête A (2015). Diabetes and immune thrombocytopenic purpura: a new association with good response to anti-CD20 therapy. Pediatr Diabetes.

[12] Quintana L, Paniagua JA, Gil-Contreras D (2010). Improving type 1 diabetes after treatment of immune thrombocytopenia with rituximab: killing two birds with one stone. Diabetes Care.

